# Mobilisation of Al, Fe, and DOM from topsoil during simulated early Podzol development and subsequent DOM adsorption on model minerals

**DOI:** 10.1038/s41598-021-99365-y

**Published:** 2021-10-05

**Authors:** Agnes Krettek, Thilo Rennert

**Affiliations:** Department of Soil Chemistry and Pedology, Institute of Soil Science and Land Evaluation, University of Hohenheim, 70593 Stuttgart, Germany

**Keywords:** Biogeochemistry, Environmental sciences

## Abstract

Podzols are characterised by mobilisation of metals, particularly Al and Fe, and dissolved organic matter (DOM) in topsoil horizons, and by immobilisation in subsoil horizons. We mimicked element mobilisation during early podzolisation by irrigating the AE horizon of a Dystric Arenosol with acetic acid at different flow velocities and applying flow interruptions to study rate-limited release in experiments with soil cylinders. We used eluates in batch experiments with goethite and Al-saturated montmorillonite to investigate DOM reactivity towards minerals. Both the flow velocity and flow interruptions affected element release, pointing to chemical non-equilibrium of release and to particles, containing Fe and OM mobilised at larger flow velocity, characteristic of heavy rain or snowmelt. Based on chemical extractions, the source of mobilised Al and Fe, the vast majority of which was complexed by DOM, was no oxide phase, but rather organic. Rate limitation also affected the composition of DOM released. Carboxyl and phenolic species were the most important species adsorbed by both minerals. However, DOM composition affected the extent of DOM adsorption on goethite more distinctly than that on montmorillonite. Our findings evidence that the intensity of soil percolation affects quantitative and qualitative element release during early podzolisation and adsorptive DOM retention in subsoil horizons.

## Introduction

Cool and humid climate impedes the decomposition of litter in soil and promotes the formation of water-soluble organic acids^1,2^ that are effective weathering agents of primary minerals and essential for the formation and development of Podzols^3–5^. Podzols are soils in which soil organic matter (SOM), iron (Fe), aluminium (Al), among other elements, are transformed, mobilised, transported, and partially immobilised in the subsoil^1,2^. As a result, a bleached eluvial horizon (e.g. EA or E) is formed on top of mostly several blackish to reddish-brown illuvial B horizons enriched in SOM (Bh) or metal oxides (Bs) or both^1,2,6^. We use the general term ‘oxides’ collectively for oxides, hydroxides, and oxyhydroxides. Podzols commonly develop under vegetation with acidic litter (e.g. coniferous forest) that releases large quantities of water-soluble organic acids^1,2^. As shown by laboratory experiments, natural organic acids accelerated weathering, e.g. dissolution of feldspars by factors of 2–3.5^4^. Due to their complexing properties, organic acids function as carrier of Al and Fe ions and contribute to their translocation in soil^7,8^. In eluvial horizons of podzolised soils, up to 80% of soluble Al was organically bound, emphasising the outstanding importance of organic complexation as a mechanism of Podzol development^9,10^. Carboxyl and phenolic OH groups were the most important functional groups^11,12^ to complex Al and Fe ions. Percolating water transported these complexes to depth, which is facilitated by highly permeable or coarse-textured parent material, from which Podzols mainly develop^1,2^. In a column experiment with extracts of Scots pine, organic acids proved to complex and translocate Al and Fe, thus contribute to the formation of AE and E horizons^13^.

Growing evidence from laboratory column studies with, for instance, a forest-floor extract percolating goethite-coated quartz sand^14^, demonstrated the paramount importance of rate-limitation for the mobility of DOM and its subsequent transport, i.e. release is no continual equilibrium process, but controlled by kinetics. Thus, with increasing residence time of the solution, dissolved organic carbon (DOC) concentrations increased due to desorption or dissolution^15^ at no-flow conditions so that DOM was increasingly mobilised after resuming the water flow. Consistently, DOM translocation under field conditions was particularly pronounced during snowmelt, when large quantities of water rapidly seeped through soil^16^. Accordingly, release and translocation of Al and Fe associated with DOM during Podzol development can be assumed rate-limited.

Accumulation of SOM, Al, and Fe in illuvial subsoil horizons is a principal feature of Podzols^17^, suggesting immobilisation of elements from overlying horizons. One process of immobilisation is precipitation of organically complexed Al and Fe as oxides because the carbon (C) to metal ratio decreases when additional metals are complexed during their passage downward the profile so that initially negatively charged complexes are neutralised^1^. This process is promoted by microbial degradation of the organic ligand^1,8^. Furthermore, cations may flocculate organic Al and Fe complexes in the subsoil^2^, and Al may precipitate with silicon (Si) as a short-range-ordered aluminosilicate^18^. Retention of organic substances in the subsoil may also be facilitated by adsorption on mineral surfaces^19,20^. In acidic soils, Fe oxides are suggested to be more efficient in DOM adsorption than clay minerals^21^. Transmission and diffuse reflectance infrared Fourier transform (DRIFT) spectra indicated the formation of Fe-carboxylate bonds by ligand exchange on the positively charged surface of goethite^21,22^. It remains unclear whether and to what extent the varying availability and changes in DOM composition due to rate-limited mobilisation and translocation from Podzol topsoil horizons influence adsorption of DOM on Fe oxides and clay minerals in the subsoil.

It is widely agreed that litter DOM actuates mobilisation of SOM and metals in mineral topsoil horizons during the formation of Podzols^1,2,7^, and that mobilisation may be a rate-limited process as shown in both field and laboratory studies^14–16^. However, the influence of rate-limited release on the speciation of Al and Fe, and the qualitative composition of DOM mobilised during early Podzol development remains unclear, as it was not systematically studied under controlled conditions yet. In this study, we triggered mobilisation of dissolved metals from an AE horizon that revealed morphological features of early Podzol development by irrigating soil cylinders. For irrigation, we used acetic acid, which is among the water-soluble organic acids present in litter-layer DOM^23,24^, as a defined model substance. These acids, percolating from the litter layer into the mineral topsoil are known to enhance weathering and translocation of metals^7–9^, thus driving podzolisation. We hypothesise that Al, Fe, and DOC eluate concentrations depend on the contact time between solution and solid material. Hence, irrigation interruptions and varying the flow velocity by a factor of 10 will significantly affect eluate concentrations^15^, and may also affect the DOM composition. Further, we tested the reactivity (estimated via adsorption on minerals) of DOM mobilised from the AE horizon in a series of batch experiments with goethite and Al-saturated montmorillonite as models of secondary subsoil minerals. We used these minerals, as they differ in their affinities for different organic species, naturally present in DOM^21^. We hypothesise that varying DOM composition, as the consequence of rate-limited release from the topsoil, will become manifest in varying compositions of the DOM adsorbed. The composition of adsorbed DOM will furthermore depend on the mineral. Consequently, rate limitation would not only control DOM mobilisation and translocation during Podzol development, but would also affect its fate in the subsoil.

## Results and discussion

### Mobilisation of DOM, Al, Fe, and Si: release experiment

**Figure 1:**
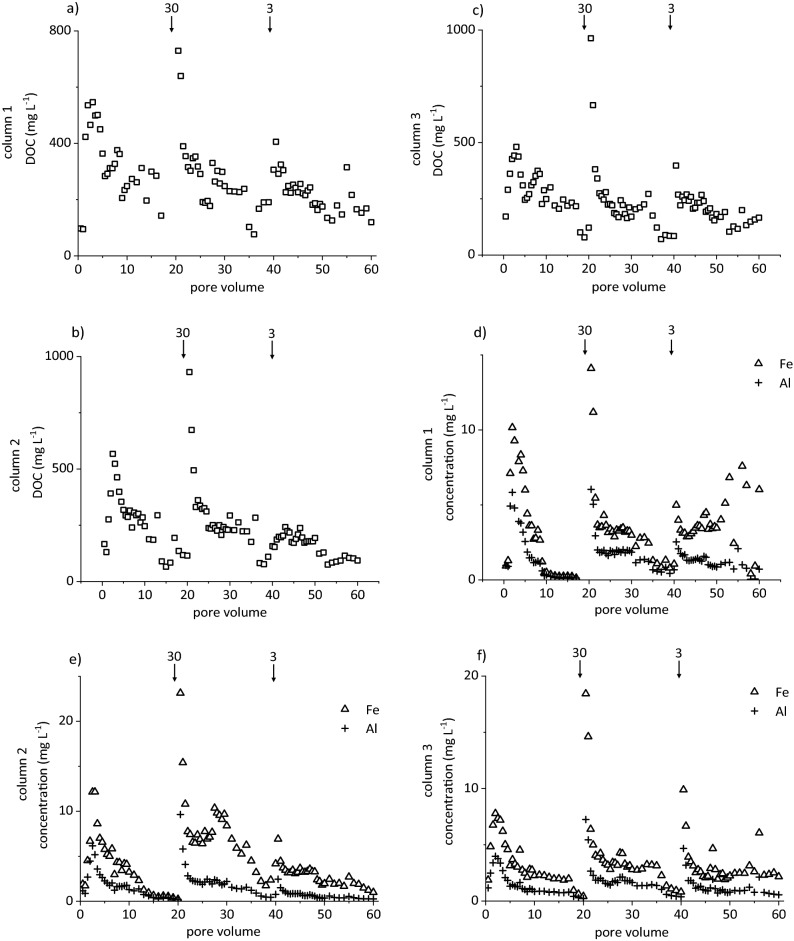
Concentrations of (**a**–**c**) dissolved organic carbon (DOC) and (**d**–**f**) Fe and Al in eluates from release experiments run at q = 1 mm h^−1^ with three soil cylinders taken from the AE horizon of a Dystric Arenosol. Arrows indicate flow interruptions and their duration in days.

**Figure 2:**
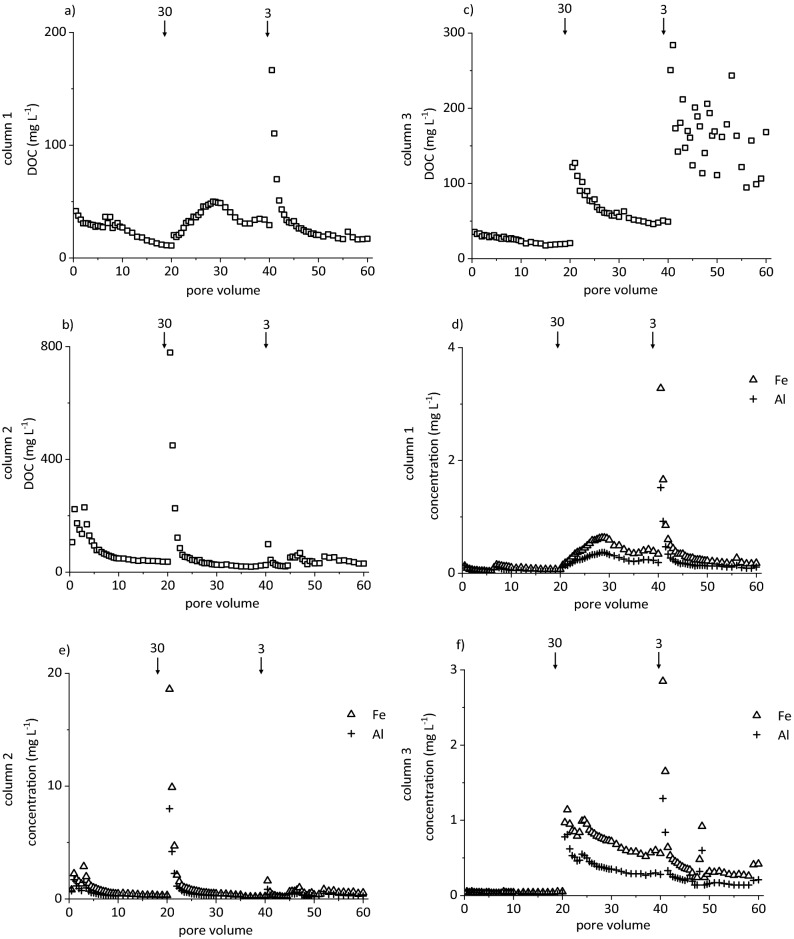
Concentrations of (**a**–**c**) dissolved organic carbon (DOC) and (**d**–**f**) Fe and Al in eluates from release experiments run at q = 10 mm h^−1^ with three soil cylinders taken from the AE horizon of a Dystric Arenosol. Arrows indicate flow interruptions and their duration in days.

After passing the soils, eluate concentrations of DOC, Al, Fe, and Si exceeded those in the initially applied acetic acid (DOC 30 mg L^−1^; Al, 10 µg L^−1^, Fe, 20 µg L^−1^; Si, 120 µg L^−1^), partially by several orders of magnitude (Figs. 1, 2; Supplementary Fig. S1). The DOC, Al, and Si concentrations in the eluates of the slow run (flow velocity q = 1 mm h^−1^) did not significantly differ among the three soil cylinders. However, Fe concentrations in eluates of cylinder 2 significantly exceeded those of the other two. Cylinders of the fast run (flow velocity q = 10 mm h^−1^) showed overall greater variability of Al, Fe, and DOC concentrations with significant differences between cylinders 1 and 3. Additionally, cylinder 3 of the fast run exhibited significantly lower Si concentrations than the other two. Differences in element elution between the experiments under identical conditions may be attributed to naturally variable structure, aggregation, and element contents of the soil cylinders^25^. Concentration patterns in the slow run were similar in all replicates. Larger differences in the fast run may point to the stronger tendency for preferential flow, as physical non-equilibrium conditions are enforced at higher flow velocity^26^, or to greater release of particles from soil by hydrodynamic shear or abrasion^27^ or both, resulting in less reproducible concentration patterns in parallel replicates. However, we consider preferential flow less important in our study, as the extremely sandy horizon (98% sand)^28^ did neither show aggregation, nor textural change, nor burrows by earthworms, which were not present in the acidic soil. Furthermore, preferential flow in soil demands a minimum clay content of 8%^29^ and a ratio of the clay and organic C contents > 0.35^30^. For the horizon under study, the latter ratio is 0.017, and the clay content is 0.2%^28^. Both numbers are at least one order of magnitude smaller than the threshold values.

The flow velocity had a significant effect on eluate DOC, Al, Fe, and Si concentrations, pointing to rate-limited release, although the concentrations were not normally distributed. The concentrations of DOC, Al, and Fe in the eluates of the slow run (Fig. 1a,b) were substantially larger than in the fast run (Fig. 2a,b), typical of rate-limited mobilisation of (in)organic compounds from topsoil during podzolisation^16^. The same applied to Si concentrations (Supplementary Fig. S1). In the initial irrigation phase, mobilisation initiated right after the start of irrigation or at the latest after the first pore volume (PV; cylinder 1). There, the release of Al, Fe, Si, and DOC corresponded to a first flush of an easily available fraction^15,26^. The pH ranged from 4.6 to 5.7 in the first PV of the slow run, dropping rapidly to < 4, i.e. approaching the pH of acetic acid used for irrigation. After each flow interruption in the slow run, the element concentrations were larger than before, also indicating rate-limited release. At no-flow conditions, the residence time of acetic acid increased, and dissolved species accumulated after desorption or dissolution^15^. Hence, the concentration increased while the pH decreased by 0.4–0.5 units after resuming the flow as compared to the concentration before the flow interruption. In the fast run, this behaviour was only evident in tendency. Accordingly, pH variations were rather unsystematic at 4.6 ± 0.4. Concentration differences before and after a flow interruption increased with increasing duration of the interruption in the slow run (Fig. 1), but showed no clear pattern in the fast run (Fig. 2). This coincided with previous studies reporting the independence of particle mobilisation of the duration of flow interruptions^27^. Accordingly, the relative concentrations of mobilised colloids (Supplementary Fig. S2) showed the same pattern and trends as Fe and DOC concentrations. This pattern was particularly evident in the fast run, but also in the slow run, particles were washed out, particularly after flow interruptions. Correspondingly, extended no-flow periods followed by high-intensity rain events promoted the mobilisation of particles from 0.7 to 200 µm as studied by zero-tension lysimeters^31^. In the slow run, Fe and DOC concentrations showed rate-limited mobilisation, but we cannot exclude additional physical mobilisation of Fe- and C-containing particles. However, the predominant mechanism of mobilisation of Fe and DOM in (coarse-textured) topsoil horizons may vary, with physical particle mobilisation during heavy rain events or snowmelt, and chemical release processes during phases of slower percolation of the soil solution.

Major anions of the eluates were very likely organic, as derived from average DOC concentrations of 251 ± 15 and 63 ± 23 mg L^−1^ in the slow and fast run, respectively. Inorganic anions were sulphate (SO_4_^2−^), nitrate (NO_3_^−^), and chloride (Cl^−^) with maximum concentrations of 4, 5.8, and 3.3 mg L^−1^ at the beginning of the experiment, but they quickly dropped below the detection limit. According to calculations with the Stockholm Humic Model, 98–100% of the eluted Al was present in organic complexes (e.g. complexed with deprotonated DOM) irrespective of the flow velocity. Only one solution, the DOC concentration of which was less than 50% of the all other solutions, contained 91% of Al in organic form and 9% as free Al^3+^ ion. The Fe eluted was always present in organic complexes.

**Figure 3:**
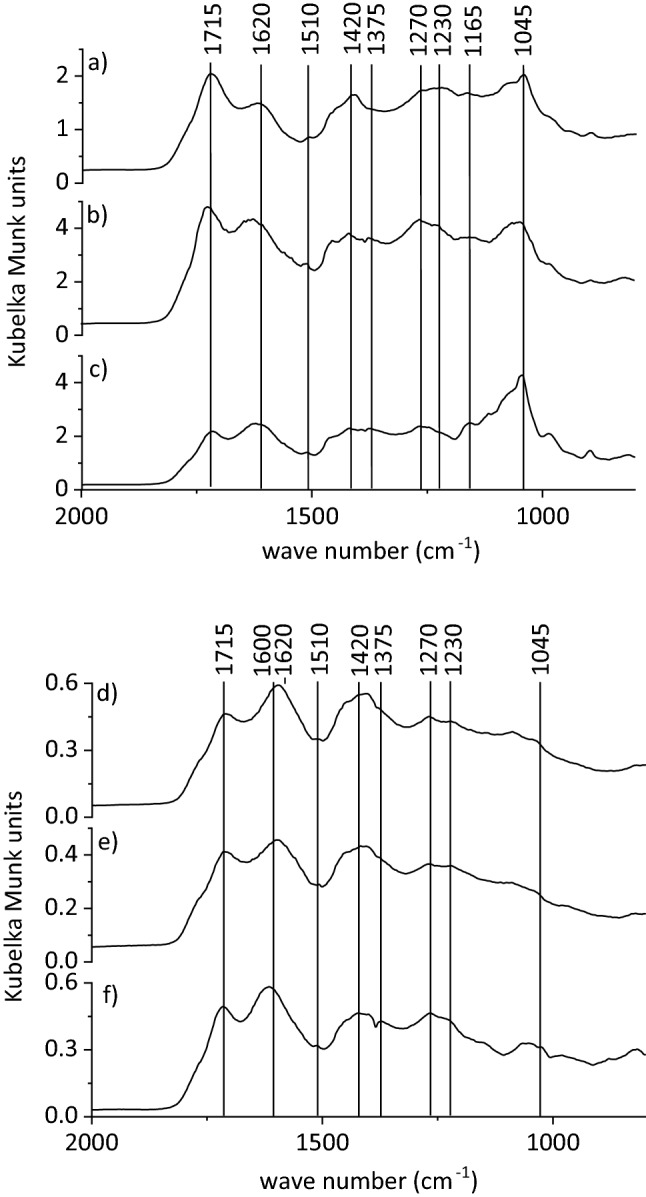
Exemplary partial diffuse reflectance infrared Fourier transform spectra of freeze-dried eluates taken from the slow (**a**–**c**) and fast run (**d**–**f**) after the first irrigation phase (**a**,**d**), after resuming the flow after 30 days of flow interruption (**b**,**e**), and after resuming the flow after 3 days of flow interruption (**c**,**f**).

DRIFT spectra of freeze-dried eluates of the slow run revealed a distinct aliphatic band at 2930 cm^−1^ and bands at 1715, 1620, 1510, 1420, 1375, 1270, 1230, 1165, and 1045 cm^−1^ (Fig. 3a–c). The bands at 1715, 1230, and 1165 cm^−1^ indicated carboxyl groups^32–34^ in protonated form, and in deprotonated form at 1620 and 1375 cm^−1^^33,35^. The band at 1620 cm^−1^ might also point to aromatic C, but the lack of a shoulder at 1580 cm^−1^ characteristic of aromatic species indicated the dominant presence of carboxylate^32^. However, C=C bonds and C–H bending of aromatic species were also evidenced by bands at 1510 and 985 cm^−1^, respectively^35,36^. Bands at 1375 and 1165 cm^−1^ may also be assigned to C–O stretching of phenolic groups and polysaccharides^35,37^, respectively. The abundance of phenolic groups was confirmed by bands at 1270 and 1420 cm^−1^^35,38^ while the band at 1045 cm^−1^ was attributed to C–O stretching vibrations of polysaccharides^32,33^. With samples taken in the course of the experiments, bands at 1045, 985, and 895 cm^−1^ became more intense and sharper, which might mirror release of dissolved or particulate Si^35^. This corroborated with Si concentrations of eluates of the slow run, which slightly increased during irrigation, but was not evident in the fast run, where, however, the same patterns of absorption bands were observed. Therefore, sharper and more intense bands at 1045 cm^−1^ rather indicated increasing proportions of polysaccharides^39^ and suggested its increasing depletion from soil. Polysaccharides adsorb more weakly than, for instance, aromatic species^40,41^ and are more easily mobilised. In the fast run, bands of carboxyl groups (1715, 1620, and 1420 cm^−1^) were dominant, while all other bands (solutions from the slow run) were mostly expressed as small humps or shoulders (Fig. 3d–f). This indicated that acetic acid used for irrigation was a dominant compound in eluates from the fast run so that little additional SOM was solubilised from soil, and confirming a more physical mobilisation of particles as the principal mechanism here. Consistent with distinctly larger DOC concentrations, more differentiated spectra of samples from the slow run and the presence of additional organic species such as phenolic species and polysaccharides indicated chemical rate-limited mobilisation. Thus, the specificity of the flow had a considerable impact on the type, composition, and amount of the mobilised organic species.

After irrigation with acetic acid, the soil in all six cylinders was strongly depleted in C (Table 1) relative to the initial content of the AE horizon (93.9 mg kg^−1^). Soil material from cylinders of the slow run had significantly lower C contents (42.8 ± 1.4 mg kg^−1^) than those of the fast run (57.6 ± 3.0 mg kg^−1^). This was consistent with larger DOC concentrations in eluates from the slow (Fig. 1a) than from the fast run (Fig. 2a), and further evidence of increasing SOM mobilisation with increasing mean residence time of acetic acid within the cylinder. Additionally, soil material of all cylinders was depleted in Fe and Al extracted by dithionite (Fe_d_, Al_d_), oxalate (Fe_o_, Al_o_), and citrate (Fe_c_, Al_c_; Table 1), which differed significantly between the fast and the slow run (except for Fe_d_). Contents of C, Fe_d_, Fe_o,_ Fe_c_, Al_d_, Al_o_, and Al_c_ were in line with previous findings on more progressively podzolised topsoils from the same study area^28^ indicating that the experiments were carried out under rather realistic conditions. All cylinders were relatively more strongly depleted in Al_d_ (43–60%) than in Fe_d_ (10–15%), particularly in the slow run, indicating rate-limited dissolution of Fe oxides, releasing Fe ions together with structurally incorporated and adsorbed Al ions. The molar Fe_d_:Al_d_ ratio increased from 1.55 (initial soil) to 2.63 after the fast run, and to 3.15 after the slow run (average of the replicate cylinders), indicating greater solubilisation of Al in the course of the experiments. Similarly, we detected increasing Fe:Al ratios for oxalate and citrate extraction [oxalate, 1.11, 1.41, 2.35; citrate, 1.15, 1.26, 2.23 (for initial contents, after the fast run, after the slow run, respectively)]. The larger mobility of Al compared to Fe can be attributed to stronger complexes that Al forms with acetate than Fe^42,43^. Then, relatively more Al than Fe is mobilised and leached from the topsoil during the course of podzolisation, while the Fe released may be transferred to a different fraction and transported more slowly from the eluvial horizon. Consistent with our experimental results, the molar Fe_d_:Al_d_ ratios of topsoil horizons of four Arenosols in the study area with increasing degree of podzolisation amounted to 1.85, 3.47, 3.69, and 3.22^28^, indicating almost the same pattern of selective metal depletion in the field and under laboratory conditions. The molar Al_d_:(Al_d_ + Fe_d_) ratios calculated for the initial material, that after the fast run and after the slow run, were 0.39, 0.28, and 0.24, may point to the initial presence of rather poorly crystalline Fe oxides such as ‘soil goethites’ or ferrihydrite with a large degree of Al substitution^44^, which were successively depleted in Al. The Fe_o_:Al_o_ and Fe_c_:Al_c_ ratios of these Arenosol topsoil horizons followed the same trend. However, dithionite-extractable Al and Fe were less depleted on a mass basis than oxalate- and citrate-extractable Al and Fe (Table 1). This indicated that solubilisation of Al and Fe from Fe oxides did not control overall metal release. Instead, the topsoil lost more Al and Fe in absolute numbers from oxalate- and citrate-soluble sources. We suggest a joint organic source of Al and Fe solubilised by the two extractants, as previously reported particularly for Podzols^28,45,46^. Dissolution of Fe oxides by citrate as used for extraction is negligible^46^. Concentrations of Al in eluates in the course of the experiment reflected rate-limited Al release from soil. However, this was not evident for the depletion in Al_o_ and Al_c_, as their contents showed little or no differences between the two flow velocities. This indicated the presence of a readily available organic Al source, extractable with both oxalate and citrate, which was predominantly solubilised and depleted during irrigation. Here, the combination of separate oxalate and citrate extraction facilitated the assignment of Fe_o_ and Al_o_ to organic forms and prevented misinterpretation^46^. The slight decrease in the Fe_c_:Fe_o_ and Al_c_:Al_o_ ratios of soil in the sequence initial soil—after fast run—after slow run (Fe, 1–0.88–0.86; Al, 1–0.99–0.9) indicated that oxalate-extractable metals did not exclusively originate from organic forms (Fe less from organic forms than Al), but also from poorly crystalline oxalate-soluble minerals. The Fe_o_:Fe_d_ ratios in the sequence initial soil—after fast run—after slow run (0.5–0.26–0.39) might reflect the preferential mobilisation of organic Fe-containing particles or poorly crystalline oxides, which are smaller than more crystalline ones, and thus more easily mobilised at fast flow.

**Table 1:** Contents of carbon (C), nitrogen (N), and dithionite-, oxalate-, and citrate-extractable Fe and Al (indicated by subscripts d, o, and c) of soil material from the AE horizon of a Dystric Arenosol before (Initial) and after irrigation with acetic acid at q = 10 mm h^−1^ (Fast) and q = 0.01 mm h^−1^ (Slow) in triplicate (C1–C3).

		C	N	Fe_d_^d^	Fe_o_^d^	Fe_c_^d^	Al_d_^d^	Al_o_^d^	Al_c_^d^
		g kg^−1^		mg kg^−1^					
Dystric Arenosol	Initial	93.9	4.3	4725	2150	2165	1235	940	945
Fast	C1	60.8	2.7	3887	994	892	787	292	334
	C2	53.6	2.5	3909	1000	908	705	346	349
	C3	58.4	2.7	3765	1129	961	687	329	387
Slow	C1	44.8	2.1	3959	1466	1398	676	265	321
	C2	41.7	1.9	3999	1677	1526	607	233	341
	C3	42.0	1.9	3780	1261	1034	553	256	290

Prior to the experiments, the soil did not contain crystalline minerals identifiable by X-ray diffraction (XRD) apart from quartz, even in the fraction < 6.3 µm (Supplementary Fig. S3). This was expectable given the sandy texture (98% sand). After irrigation, small reflexes at 7 and 10 Å appeared, pointing to kaolinite and illite, respectively. This indicates that X-ray amorphous substances such as short-range-ordered aluminosilicates, Fe oxides, or OM initially covered the small amounts of clay minerals present and masked their XRD reflexes, but were dissolved by acetic acid in the course of the release experiments. Accordingly, XRD patterns of topsoils in the study area with increasing degree of podzolisation showed increased reflexes at 10 Å (Supplementary Fig. S3, samples P3–P5) relative to the less podzolised AE horizon (P1) used for the experiments in the present study. We interpret the more distinct reflexes of samples P3–P5 as the result of podzolisation, which includes weathering and dissolution of X-ray amorphous substances to increasing extent. Thus, irrigation with acetic acid in the laboratory induced mobilisation of SOM, Al, and Fe similar to that under natural field conditions. Soil material after the fast run further exhibited slight reflexes at 10–13 Å pointing to hydroxy-interlayered minerals. These result from weathering of primary silicates in a geochemical milieu typical of early Podzol development, i.e. pH approximately > 4.5, which prevents acidic dissolution of 2:1 layers of phyllosilicates^47^. Despite the limited duration of the experiment, mimicked weathering of the topsoil showed that masking phases were easily removed. These may correspond to the readily available organic Al and Fe (extracted by oxalate and citrate) described earlier, which were predominantly mobilised in the experiment and constituted the depleted sources of the solid material.

### Adsorption of mobilised DOM on goethite and Al-saturated montmorillonite: batch experiments

**Figure 4:**
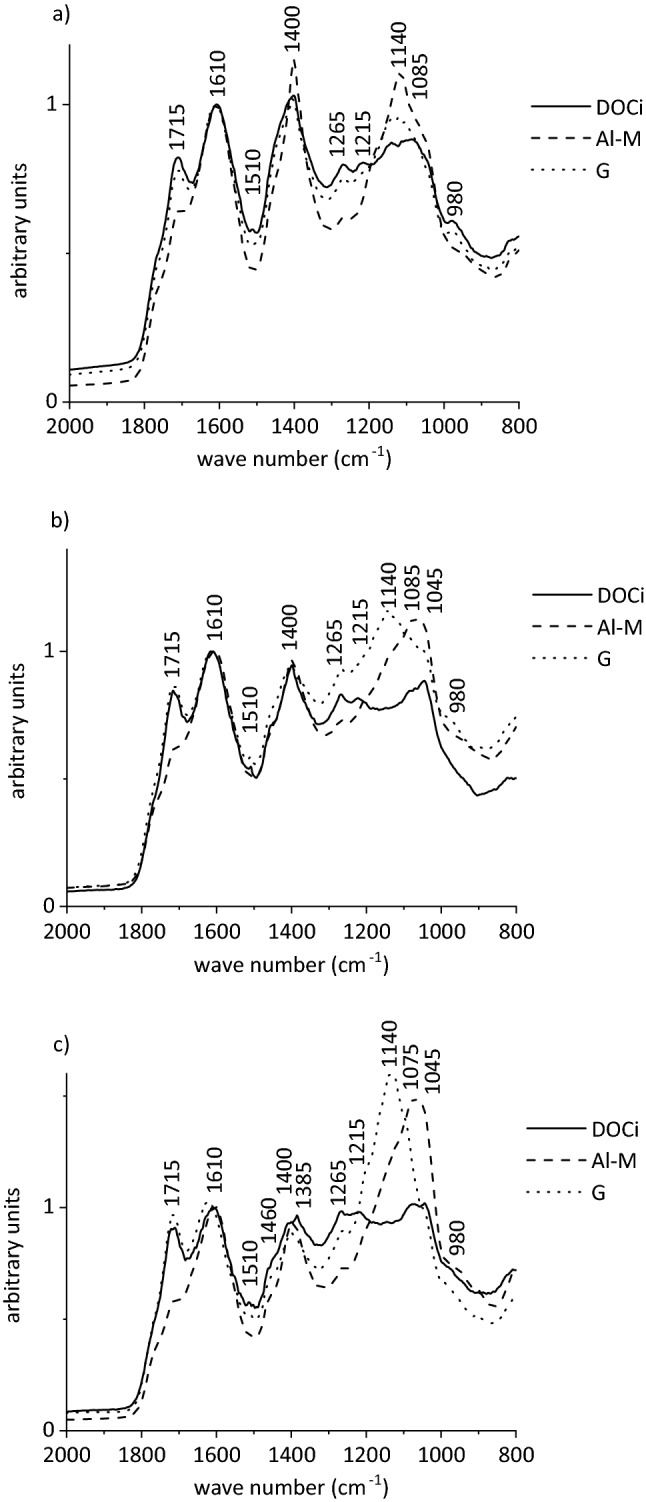
Exemplary partial diffuse reflectance infrared Fourier transform spectra of freeze-dried composite solutions taken after (**a**) 1–10, (**b**) 11–20, and (**c**) 31–40 pore volumes of the slow run prior to adsorption (DOCi) and of filtrates after adsorption experiments with Al-montmorillonite (Al-M) and goethite (G). All spectra were normalised to the band at 1610 cm^−1^.

Combining and processing several eluates into composite solutions for the adsorption experiments induced qualitative changes compared to the original eluates of the release experiments (Fig. 4a–c). On the one hand, these were slight band shifts at 1620, 1420, 1270, 1230, and 985 cm^−1^ in spectra of the eluates to 1610–1600, 1400, 1265, 1215, and 995/980 (slow/fast) cm^−1^ in spectra of composite solutions, respectively. On the other hand, bands of deprotonated carboxyl groups at 1610 and 1400 cm^−1^ increased while those of protonated carboxyl groups at 1715, 1215, and 1165 cm^−1^ decreased according to the increase in pH to 4. Besides, bands at 1045 cm^−1^ were less intense and sharp or gone compared to eluates of the release experiments (Fig. 3) pointing to the removal of particles > 0.45 µm containing DOM or Si or both by filtration.

**Figure 5:**
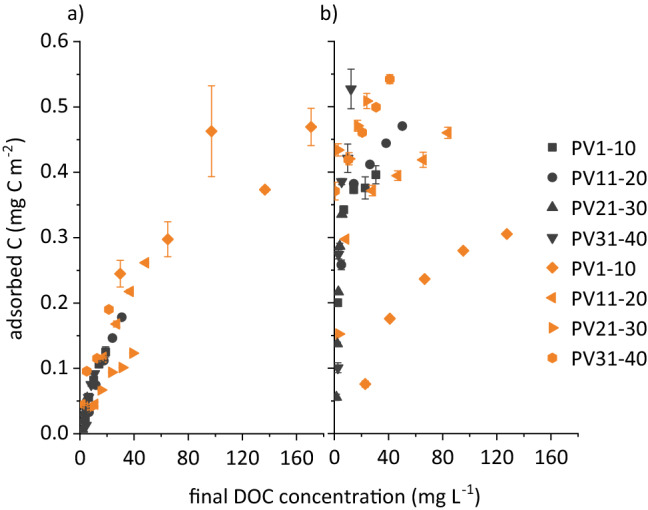
Adsorbed C related to the specific surface area of (**a**) Al-montmorillonite and (**b**) goethite against the final DOC concentration of composite solutions from release experiments at q = 1 mm h^−1^ (orange, slow run) and q = 10 mm h^−1^ (black, fast run). Error bars represent standard errors of duplicates.

Normalised to the SSA of the mineral (goethite, 14.7 m^2^ g^−1^; Al-montmorillonite, 92.9 m^2^ g^−1^), DOC adsorption on goethite was more pronounced than on Al-montmorillonite, except for the composite solution taken after 1–10 PVs of the slow run (Fig. 5). When plotting the entire adsorption data of Al-montmorillonite from eight composite solutions, it appeared as if the eight solutions behaved like one, particularly the solutions from the fast run (Fig. 5a). Obviously, all eight composite solutions provided sufficient preferentially adsorbing organic species, irrespective of the initial DOC concentration and DOM composition, and thus the flow regime. Irrespective of the mineral adsorbent and the adsorptive DOM solution, absorption bands at 1140, 1085, and 1045 cm^−1^ increased in spectra of filtrates after adsorption (Fig. 4), indicating that polysaccharides were excluded to larger extent from adsorption than other species and accumulated in the supernatants. However, apart from the experiments shown in Fig. 4a, exclusion of polysaccharides was more pronounced for goethite. Larger and hydrophobic molecules may have more functional groups that bind simultaneously on the Al-saturated montmorillonite surface by cation bridges and occasionally by ligand exchange at hydroxylated edges. As exemplarily shown in Fig. 4, Al-montmorillonite preferentially adsorbed organic species characterised by carboxyl, phenolic OH and other aromatic groups, as the intensities at 1715, 1280–1240, and 1510 cm^−1^ decreased after adsorption for all variants with Al-montmorillonite involved. Saturation of montmorillonite surfaces with Al^3+^ promoted adsorption by forming cation bridges^21^. Accordingly, species with carboxyl groups were adsorbed, even in protonated state, as reflected by the decrease in absorption at 1715 cm^−1^ after adsorption. This was a further indication of adsorption by ligand exchange, which was also expressed by increasing pH to 5.5–6 of the filtrates after adsorption. As we normalised the spectra of the freeze-dried filtrates after adsorption to the band at 1610 cm^−1^, we cannot approximate the extent of adsorption of deprotonated carboxyl groups. However, as this band was prominent in any spectrum, normalisation enabled us to approximate the contribution of other functional groups to overall DOM adsorption, potentially varying between the variants. Generally, our findings are in line with the observation that carbohydrates and N-rich compounds accumulate in the soil solution of Podzol subsoil horizons, while compounds rich in carboxyl and aromatic groups are preferentially adsorbed on soil minerals^32,48^.

The extent of DOC adsorption on goethite differed marginally between the four solutions from the fast run (Fig. 5b), pointing to very similar DOM species available for adsorption (Supplementary Fig. S4). Consistently and as mentioned before, we detected very similar compositions of the original eluates in the fast run. Consequently, the extent of DOM adsorption from the composite solutions from the fast run was very similar, and the strong increase in adsorbed DOC with increasing equilibrium concentration reflected the large affinity of the adsorptive DOM towards the goethite surface. However, the extent of adsorption varied between the composites taken from the slow run (Fig. 5b), with more DOC adsorbed from composite solutions taken from PVs 21–30 and 31–40 than from PVs 11–20 and, fewest, from PVs 1–10. Comparing the composition of the solutions from the slow run prior to adsorption (Fig. 4), it is obvious that the intensity at 1715 cm^−1^, relative to the band at 1610 cm^−1^, to which all spectra were normalised, decreased in the sequence PVs 31–40 > PVs 11–20 > PVs 1–10, while that at 1265 cm^−1^ increased. The latter is characteristic of phenolic OH groups, which are particularly important for adsorption on Fe oxides, rather than on aluminol groups^32,38^. We adjusted the pH of the composite solutions to 4, i.e. we decreased the degree of protonation of carboxyl groups, e.g. those of acetate with a pK_a_ value of 4.8. Protonation was more strongly decreased with the composites of the slow run, which had initial pH values of 3.6–3.8, while composites from the fast run already had approximately pH 4. The final pH after adsorption on goethite increased by 0.2–0.3 units when using the solutions taken after 1–10 and 11–20 PVs exchanged from the fast run as adsorptive, but did not increase in the other variants, indicating that ligand exchange did not play a major role. Consistently, absorption at 1715 cm^−1^, indicating protonated carboxyl groups did not decrease after DOM adsorption (Fig. 4). However, absorption at 1380 cm^−1^, indicating deprotonated carboxyl groups^54^, decreased, confirming preferential adsorption of deprotonated rather than protonated carboxyl groups (1715 cm^−1^) on goethite. Figure 5b clearly shows that in our experiments the goethite surface was far from being covered, as the adsorbed amounts did not converge. Consequently, DOM adsorption on goethite, rather than on Al-montmorillonite, depended to larger extent on the composition of the adsorptive solution, and thus on the flow regime. Similarly, DOM from water extracts of Oa horizons was more distinctly fractionated by adsorption on montmorillonite than by goethite^21^. In that study, goethite had a lower affinity for polysaccharides, but adsorbed more DOM with a larger molecular mass, e.g. aromatic species, compared to montmorillonite, which is consistent with our findings.

The results of our adsorption experiments confirmed that DOM leaching from A horizons during early formation and development of Podzols into subsoil horizons consists of organic species that are capable of adsorbing on mineral surfaces, even though differentiated by the type of mineral^20,49^. Consequently, SOM in illuvial B horizons may at least partially derive from adsorbed DOM^28,41,50^. Our previous findings on more progressively podzolised sandy soils in the study area showed increased accumulation of SOM in aggregates and coatings of SOM on sand grains intimately associated with Fe phases and increasing amounts of Al in illuvial subsoil horizons^28^. Although DOM adsorption on mineral surfaces in aggregates/accounting for coatings could not be excluded, SOM, Al, and Fe tended to be in organic associations to larger extent, i.e. as flocculated organic precipitates. Particularly in very sandy substrates, the availability of mineral surfaces for DOM adsorption is limited. On the other hand, SOM present in the fraction < 63 µm of these soils was enriched in carboxylate and aromatic C, confirming their preferential removal from solution by adsorption, as found in the present study, particularly for Al-montmorillonite.

## Conclusions

We could verify our first hypothesis that release of DOM, Al, and Fe during an early stage of Podzol formation is rate-limited. The processes affecting the amount and speciation of the compounds released differed as a function of the irrigation intensity. Accordingly, mobilisation of Fe and DOM may be dominated by physical particle mobilisation during heavy rain events or snowmelt, while at slower percolation, chemical release processes may dominate, resulting in mobilisation of Al and Fe ions as organic complexes. Based on soil characterisation after the podzolisation experiment, release and depletion of Al and Fe from the topsoil was decoupled. Iron depletion tended to be slower than that of Al, which formed stronger complexes with organic ligands, resulting in species that were easily translocated. Nonetheless, the depletion of Al and Fe from all soil fractions susceptible to extraction with dithionite, oxalate, and citrate was rate-limited, consistent with field data. Extraction data did not point to preferential release of Al and Fe from Fe oxides, but from organic sources, which points to a yet unknown aspect of initial podzolisation, which, however, requires analytical evidence beyond extraction. Our results confirmed that oxalate extraction alone is unsuitable for a quantification of poorly crystalline Al and Fe species, as long as the organic proportion of extracted metals is unknown. Rate-limitation of DOM release resulted in increased DOC concentrations after no-flow periods and qualitative differentiation, with more polysaccharides and phenolic species at moderate percolation conditions and after phases of no percolation. These findings do not only confirm the known seasonality of overall quantitative DOM translocation in Podzols, but they additionally point to variable qualitative DOM composition that affects reactivity of DOM towards mineral surfaces and thus its fate in the soil profile. The DOM released from the A horizon always consisted of carboxyl groups that adsorbed on the Al-montmorillonite by ligand exchange, i.e. irrespective of the protonation state of the carboxyl groups. However, quantitative and qualitative DOM adsorption on goethite was more distinctly affected by the composition of the adsorptive solution than Al-montmorillonite, thus by effects of rate-limited release. Hence, we could verify our second hypothesis that DOM adsorption on two types of minerals is mineral-specific and depending on DOM composition, controlled by rate-limited release, which thus affects the fate of DOM in Podzols. Related to the specific surface area of the minerals, saturation with SOM did not occur during initial podzolisation, but adsorption on goethite was more strongly affected by changes in pH, controlling the degree of deprotonation of carboxyl groups, and the general quantitative and qualitative composition of the adsorptive. We conclude that fractionation of SOM detected in Podzol subsoil horizons is at least affected by these adsorptive processes. However, further processes are involved in SOM storage in Podzol subsoil horizons, including micro-aggregate formation and preferential accumulation of carbohydrates, as recently reviewed^51^.

## Materials and methods

### Site description and sampling

We conducted experiments with the AE horizon of a Dystric Arenosol developed from Pleistocene aeolian sand (0.2% clay, 1.8% silt, 98% sand) located on the Lower Rhine Plain in NW Germany [51° 10′ 16″ N|6° 11′ 40″ E]. The horizon was strongly acidic with pH 2.7 (CaCl_2_) and about 8–10 cm thick. After removing the L and O horizons, soil cylinders (stainless steel, inner diameter 72 mm, height 61 mm, V = 250 cm^3^, six replicates) were pounded into the soil and carefully removed to maintain the integrity of the soil. General characteristics of the horizon (Table 1) were described previously^28^. We took a further three separate soil cylinders (V = 100 cm^3^) to determine the bulk density by weighing after drying at 105 °C. The bulk density was 0.84 g cm^−3^.

### Release experiments

**Figure 6:**
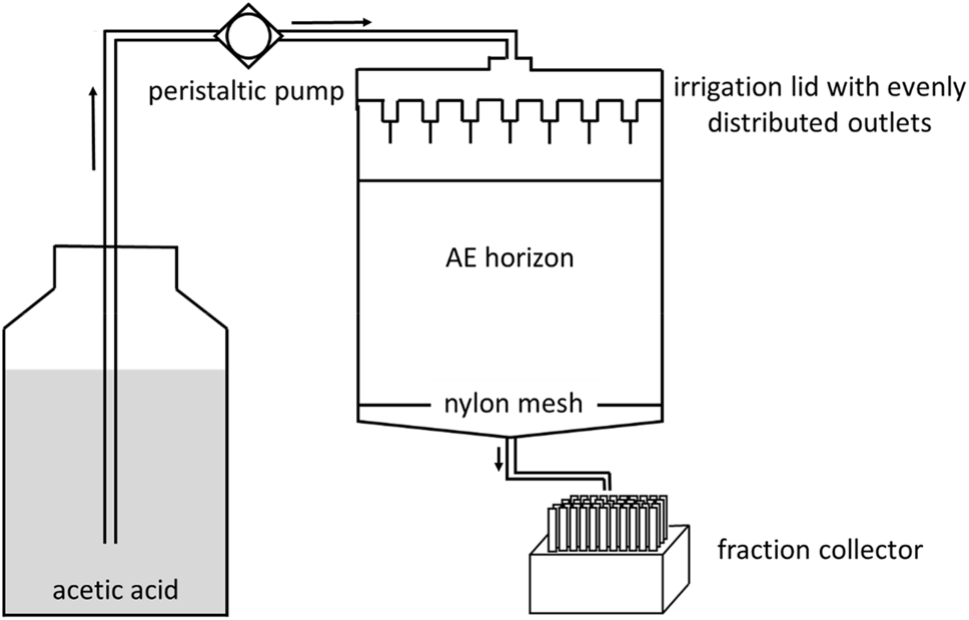
Schematic illustration of the release experimental setup.

The soil cylinders were placed in PVC pipes (inner diameter 90 mm, height 8.5 cm) and covered with a nylon mesh and an outlet funnel at the bottom (Fig. 6). A peristaltic pump (ISMATEC MCP, Cole-Parmer GmbH, Wertheim, Germany) supplied 100 µmol L^−1^ acetic acid, which is a mean concentration found in litter layers/organic horizons of Podzols^23,24^, to an irrigation unit, which was customised to fit on top of the PVC pipe. The experiments were run at two flow velocities in triplicate, with q = 10 mm h^−1^ (fast run) and q = 1 mm h^−1^ (slow run) to simulate strong leaching with large amounts of seepage water (heavy rain events or snowmelt^16^) and moderate rain, respectively. We used atmospheric boundary conditions (BC) with the soil surface as upper BC and the seepage face as lower BC. The flow velocity resulted from dividing the volumetric flow rate Q by the cross-sectional area of the cylinder. Varying q by a factor of 10 allows to observe variations in eluate-concentration patterns caused by chemical non-equilibrium^15^. Additionally, we interrupted the flow twice for 30 and 3 days to detect rate-limited release of organic and inorganic constituents. In the case of rate-limited release, their concentrations increase after resuming the flow following a flow interruption, as release at chemical non-equilibrium continues during no-flow periods, i.e. dissolved species accumulate as a function of extended residence time of the eluent^52^. Prior to and following each flow interruption, we collected the eluates during the exchange of 20 PVs altogether. We sampled 50 mL after 0.5 PVs exchanged during the first 10 PVs and increased the sampling interval to one PV for the following 10 PVs exchanged using two fraction collectors. A total of 60 PVs was exchanged for each soil cylinder.

As we needed all soil materials after the release experiments for chemical analyses, we did not determine the PV of each individual soil cylinder after the experiments, which would have included drying at 105 °C. Instead, we used an approximated PV for all soil cylinders, which we calculated from the bulk density ρ_bulk_ determined with the separate soil cylinders (0.84 g cm^−3^) and the density of the soil. As the soil material was almost completely consisting of sand (98%), we considered the density of quartz (2.65 g cm^−3^) for the mineral fraction of the soil, and 1.4 g cm^−3^ for the organic fraction. This resulted in a density of ρ_soil_ = 2.6 g cm^−3^ of the soil material, considering the initial C content (Table 1). Consequently, the porosity ε was 68.3% (ε = 100 × ρ_bulk_/ρ_soil_), resulting in PV = 170.75 ml (PV = ε × V_cylinder_).

We determined DOC concentrations by catalytic high-temperature oxidation (DIMATOC 2100, Dimatec Analysen GmbH, Essen, Germany). Analyses for Fe, Al, and Si were conducted by microwave plasma-atomic emission spectrometry (4200 MP-AES, Agilent, Waldbronn, Germany), and for inorganic anions (SO_4_^2−^, NO_3_^−^, Cl^−^) by ion chromatography (850 Professional IC Anion, Metrohm, Filderstadt, Germany). We determined eluate pH potentiometrically, and the relative colloid concentration by UV–Vis spectroscopy (Cary 50 Conc, Varian, Darmstadt, Germany) at λ = 350 nm^53^. Samples for qualitative characterisation of eluted DOM were taken at the beginning, during and at the end of each irrigation phase to trace changes during irrigation and after flow interruptions. Therefore, a subsample of 10 mL was frozen, freeze-dried, and characterised by DRIFT spectroscopy, using the external DRIFT accessory of a LUMOS infrared microscope (Bruker, Ettlingen, Germany). We mixed each sample at a ratio of 1:20 with potassium bromide (KBr). Spectra of pure KBr were recorded as background. For each sample, 200 scans were accumulated at a resolution of 4 cm^−1^ in the spectral range of 4000–600 cm^−1^. The spectra were converted to Kubelka–Munk units using OPUS 7.2 (Bruker).

Speciation in solution was assessed using Visual MINTEQ version 3.1^42^. To calculate the extent of metal complexation with DOC, we used the Stockholm Humic Model (SHM)^54^ employing a discrete-site approach. At widely different pH and equilibrium concentrations, SHM is capable of describing metal binding and competitive interactions^54^. The input parameters were pH, and the concentrations of DOC, inorganic anions, Al, Fe, and Si.

We analysed the soil material (n = 6) for total C and N contents with an elemental analyser (Vario macro EL, Elementar, Hanau, Germany) before and after the experiments, using air-dried material. In addition, we extracted the soil materials separately for Al and Fe in the entirety of Fe oxides by dithionite-citrate-bicarbonate^55^, for Al and Fe in poorly crystalline Fe oxides, short-range-ordered aluminosilicates, and partially in organic complexes by oxalate-oxalic acid in darkness^56,57^, and for Al and Fe in organic complexes by citrate ^46,58^. All analyses were conducted in triplicate. We determined Al and Fe concentrations in the extracts by MP-AES. We obtained the fine silt and clay fraction (< 6.3 µm) by centrifugation and analysed the qualitative mineral composition after the experiments by XRD using a Bruker D2 Phaser (Co-Kα radiation, U = 40 kV, I = 5 mA). Samples were milled and measured as topfill powder mounts at diffraction angles 2θ = 4°–90° with a step size of 0.02° and a counting time of 32.5 s per step.

### Adsorption experiments

Adsorption experiments were conducted with eight different composite solutions taken from the release experiments. Four solutions were taken from the fast and the slow run, respectively, by collecting 600 mL during the first irrigation period and after the first flow interruption. Solutions representing 10 PVs were combined to a composite solution i.e. from PVs 1–10, 11–20, 21–30, and 31–40 per release experiment. We filtered all composite solutions [0.45 μm cellulose-nitrate filters (Sartorius, Göttingen, Germany)], filled up to 1 L, and adjusted to pH 4 with 10% NaOH to prevent subsequent undesired dissolution of model minerals. The initial DOC concentrations of the composite solutions used for the adsorption experiments ranged from 28 to 185 mg L^−1^.

We used two minerals as models of important DOM adsorbents, including montmorillonite from a natural deposit (Erbslöh, Geisenheim, Germany) as a model of a 2:1 clay mineral potentially present in Podzol subsoil horizons of temperate and high latitude. We saturated the montmorillonite with Al^3+^ using a 1 M AlCl_3_ solution and subsequent washing with deionised water to account for Al as dominating cation in the soil solution at low pH. We used a commercially available goethite (Bayferrox 920, Lanxess, Köln, Germany) as a model of a stable Fe oxide potentially present in Podzol subsoil horizons instead of a 2-line ferrihydrite, which was partially dissolved in preliminary adsorption experiments, owing to low pH. We determined the specific surface area of the minerals by N_2_ sorption and desorption at 77 K using a Quantachrome Autosorb iQ (Anton Paar Quanta Tec Inc., USA), according to the Brunauer–Emmett–Teller equation, after degassing for at least 8 h at 333 K.

We suspended 100 mg of each mineral in 10, 20, 30, 40, or 50 mL of a composite solution and diluted to 50 mL deionised water in duplicate. The samples were shaken horizontally for 24 h (125 cycles min^−1^) at 21 °C and filtered subsequently (0.45 µm). We measured the pH of the solution before and after the adsorption experiments and analysed the filtrates for DOC. To characterise the composition of DOM removed from solution during adsorption, we freeze-dried 20 mL of the filtrates when containing > 0.3 mg C, and characterised them by DRIFT spectroscopy. We mixed each sample with KBr at a ratio of 1:20 and conducted DRIFT analyses as described before. We used the band at 1610 cm^−1^ for normalisation of all DRIFT spectra, as the band was prominent in all spectra.

We used IBM SPSS Statistics 27 for statistical analyses. Analysis of variance (ANOVA) was used to check significant differences in the elemental concentrations among eluates of both runs, and in C contents and contents of extracted Al and Fe in soil before and after the release experiments (*p* < 0.05). Levene’s test was used to test for homogeneity of variances. For post-hoc analyses, according to the homogeneity of variances, the Tukey (*p* > 0.05) or Games-Howell (*p* < 0.05) test was used to check variation of the t-test.

## Supplementary Information

Supplementary Information.
