# Supplementary material for: Mobilisation of Al, Fe, and DOM from topsoil during simulated early Podzol development and subsequent DOM adsorption on model minerals
Source: Sci Rep. 2021 Oct 5;11:19741. doi: 10.1038/s41598-021-99365-y (PMC8492631; doi:10.1038/s41598-021-99365-y)
Supplement: Supplementary file 1 — Supplementary Information. [file 41598_2021_99365_MOESM1_ESM.docx]

**Mobilisation of Fe, Al, and DOM from topsoil during simulated early Podzol development and subsequent DOM adsorption on model minerals**

**Agnes Krettek, Thilo Rennert**

Department of Soil Chemistry and Pedology, Institute of Soil Science and Land Evaluation, University of Hohenheim, D-70593 Stuttgart, Germany

Supplementary material

Figure S1: Concentration of Si in eluates from release experiments run at q = 1 mm h^-1^ (a)-c)) and q = 10 mm h^-1^ (d)-f)) with three soil cylinders taken from the AE horizon of a Dystric Arenosol. Arrows indicate flow interruptions and their duration in days.

Figure S2: Total extinction [-] at λ = 350 nm in eluates from release experiments run at q = 1 mm h^-1^ (a)-c)) and q = 10 mm h^-1^ (d)-f)) with three soil cylinders taken from the AE horizon of a Dystric Arenosol. Arrows indicate flow interruptions and their duration in days.

Figure S3: Partial X-ray diffraction patterns of fine silt and clay fractions (<6.3 µm) of the AE horizon of a Dystric Arenosol before (P1) and after irrigation with acetic acid at q = 10 mm h^-1^ (fast) and q = 1 mm h^-1^ (slow). Additional X-ray diffractograms of podzolised topsoils (P2-P5) from the study area for comparison^1^.

Figure S4: Exemplary diffuse reflectance infrared Fourier transform spectra of freeze-dried composite solutions taken after a) 1-10 pore volumes, b) 11-20 pore volumes, and c) 31-40 pore volumes of the fast run prior to adsorption (DOCi) and of filtrates after adsorption experiments with Al-montmorillonite (Al-M) and goethite (G). All spectra were normalised to the band at 1610 cm^-1^.

1. Krettek, A., Herrmann, L. & Rennert, T. Podzolisation affects the spatial allocation and chemical composition of soil organic matter fractions. *Soil Res.* **58**, 713–725 (2020).
